# Non-Invasive Brain Sensing Technologies for Modulation of Neurological Disorders

**DOI:** 10.3390/bios14070335

**Published:** 2024-07-09

**Authors:** Salman Alfihed, Majed Majrashi, Muhammad Ansary, Naif Alshamrani, Shahad H. Albrahim, Abdulrahman Alsolami, Hala A. Alamari, Adnan Zaman, Dhaifallah Almutairi, Abdulaziz Kurdi, Mai M. Alzaydi, Thamer Tabbakh, Faisal Al-Otaibi

**Affiliations:** 1 Microelectronics and Semiconductor Institute, King Abdulaziz City for Science and Technology (KACST), Riyadh 11442, Saudi Arabia; salfihed@kacst.edu.sa (S.A.);; 2 Bioengineering Institute, King Abdulaziz City for Science and Technology (KACST), Riyadh 11442, Saudi Arabia; 3 Neuroscience Center Research Unit, King Faisal Specialist Hospital and Research Centre, Riyadh 11211, Saudi Arabia; 4 Advanced Materials Institute, King Abdulaziz City for Science and Technology (KACST), Riyadh 11442, Saudi Arabia; akurdi@kacst.edu.sa

**Keywords:** non-invasive brain sensing, brain neuromodulation techniques, closed-loop neuromodulation systems

## Abstract

The non-invasive brain sensing modulation technology field is experiencing rapid development, with new techniques constantly emerging. This study delves into the field of non-invasive brain neuromodulation, a safer and potentially effective approach for treating a spectrum of neurological and psychiatric disorders. Unlike traditional deep brain stimulation (DBS) surgery, non-invasive techniques employ ultrasound, electrical currents, and electromagnetic field stimulation to stimulate the brain from outside the skull, thereby eliminating surgery risks and enhancing patient comfort. This study explores the mechanisms of various modalities, including transcranial direct current stimulation (tDCS) and transcranial magnetic stimulation (TMS), highlighting their potential to address chronic pain, anxiety, Parkinson’s disease, and depression. We also probe into the concept of closed-loop neuromodulation, which personalizes stimulation based on real-time brain activity. While we acknowledge the limitations of current technologies, our study concludes by proposing future research avenues to advance this rapidly evolving field with its immense potential to revolutionize neurological and psychiatric care and lay the foundation for the continuing advancement of innovative non-invasive brain sensing technologies.

## 1. Introduction

Research in brain sensing and neuromodulation has seen significant progress, with the emergence of a technique that offers treatment for a variety of neurological and psychiatric disorders [1]. This technique involves targeted stimulation of specific regions of the brain to trigger/inhibit specific neural circuits. Previously, deep brain stimulation (DBS) was the primary method used for neuromodulation, requiring the surgical implantation of electrodes within the brain [2]. However, non-invasive neuromodulation has gained momentum in recent years, utilizing energy sources such as ultrasound, electrical currents, and electromagnetic fields to modulate brain activity from outside the skull. This therapy is a safer and more accessible alternative to conventional DBS, eliminating the risks associated with surgery and anesthesia and making it more patient-friendly [3,4]. Non-invasive neuromodulation can be precise in targeting specific areas of the brain and has proven to be more effective in treating various conditions [3]. Non-invasive neuromodulation is a promising technique with tremendous potential to revolutionize the field of neuroscience and provide better treatment options for patients [4]. For instance, transcranial magnetic stimulation (TMS) involves placing a magnetic coil on the scalp to create a magnetic field that can stimulate specific brain areas [5,6,7,8,9]. Similarly, transcranial electric stimulation (tES) uses electrical currents applied to the scalp to regulate brain activity [10]. These techniques are less risky and more patient-friendly, as they do not require surgery or anesthesia. 

Non-invasive brain sensing and neuromodulation techniques have exhibited potential in the management of a wide range of neurological and psychiatric conditions, including anxiety, depression, chronic pain, and Parkinson’s disease (PD) [11]. However, more research is needed to understand their effectiveness and potential risks. As such, non-invasive neuromodulation remains a vital area of study for the future of neurological and psychiatric treatments [6,7,8,9]. Several subtypes of non-invasive brain sensing and neuromodulation exist, each possessing unique properties and mechanisms of action. Ultrasound neuromodulation utilizes precisely targeted sound waves to stimulate brain tissue. Electrical stimulation techniques, such as TMS and transcranial direct current stimulation (tDCS), employ weak electrical currents or magnetic fields to influence neuronal firing patterns [12]. Non-invasive optogenetic neuromodulation, though still primarily confined to the research realm, offers highly targeted control by utilizing light to activate or inhibit genetically modified neurons [13].

This study comprehensively examines the recent advancements in non-invasive brain sensing and neuromodulation. We probe into the various methods available, including ultrasound, electrical, and electromagnetic stimulation, exploring their specific mechanisms and potential for treating diverse brain disorders. We compared the aforementioned non-invasive neuromodulation techniques with a keen interest in spatial resolution and depth of penetration. Subsequently, we discuss the emerging concept of closed-loop neuromodulation systems, which offer real-time feedback and personalized stimulation based on an individual’s brain activity. Ultimately, an acknowledgment of the limitations of current non-invasive technologies and the future directions for research and development in this rapidly evolving field are discussed.

## 2. Non-Invasive Neuromodulation for Brain Disorders

**Figure 1:**
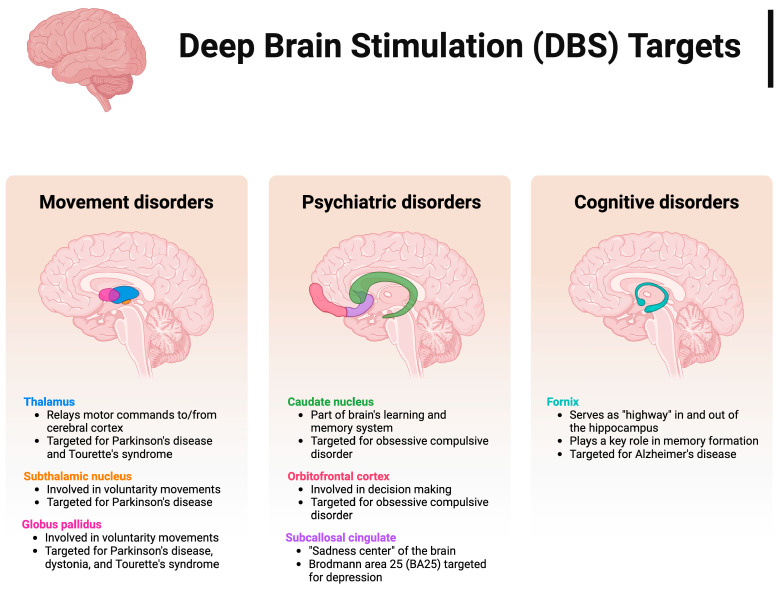
Anatomic targets of deep brain stimulation for neurological and psychiatric disorders, including movement disorders, psychiatric disorders, and cognitive disorders. Created with BioRender.com.

Even though some invasive neuromodulators, such as DBS, have proven effective in symptom treatment of multiple neurodegenerative disorders, such as obsessive-compulsive disorder (OCD), epilepsy, and Parkinson’s disease [2], invasive neuromodulation poses risks to users, including, but not limited to, brainjacking, which is the unwarranted access and control of a patient’s electronic implants [14], the operation’s high cost, and the risk of hemorrhage when implanting wires [15], which can also be a source of infection when in vivo [16]. Figure 1 shows DBS targets for various neurological conditions. In movement disorders, DBS targets the thalamus, the subthalamic nucleus, and the globus pallidus. In psychiatric disorders, DBS targets focus on areas like the caudate nucleus, orbitofrontal cortex, and subcallosal cingulate. Ultimately, for cognitive disorders, the fornix is a potential DBS target. Therefore, non-invasive brain stimulation (NIBS) has received increased research interest, which involves ultrasound, electrical, and electromagnetic stimulation [3,11]. As each tool relies on a different energy type, these tools also differ in terms of temporal resolution, spatial resolution, and depth [3,4]. Concerning the widely used types of neuromodulation, transcranial-focused ultrasound stimulation (tFUS) can target deep regions of the brain and has a higher spatial resolution than both tDCS and repetitive transcranial magnetic stimulation (rTMS). Thus, tFUS can reach subcortical regions of the brain, including the thalamus, amygdala, and hippocampus [4,17]. In contrast, rTMS, and tDCS [6,7,8,9]. There has been a significant increase in interest in utilizing non-invasive neuromodulation to target specific brain diseases and disorders such as Alzheimer’s disease (AD), PD, OCD, dystonia [18,19,20,21,22,23], and even multiple sclerosis (MS) fatigue amelioration by a tDCS system [24], with which they have been proven effective. The following sections will explore the latest advancements in current non-invasive neuromodulation techniques in brain disorder treatment. This section discusses the different brain stimulation techniques, including ultrasound, electrical, and electromagnetic stimulation. 

### 2.1. Ultrasound Stimulation

The two modes of ultrasound (US) used in neuromodulation are transcranial-unfocused ultrasound stimulation (tUS) and tFUS. Both techniques are non-invasive and acoustic-based, with which a reversible modulation of deep or superficial brain regions can be achieved [4]. Focused ultrasound aims to converge acoustic waves gained by either using a concaved transducer, a flat transducer with focusing lenses, or a phased-array transducer in which multiple, out-of-phase wave signals are sent to different parts of the transducer, achieving a narrower field-of-view (FOV) within the brain-targeted region [12]. For both US technologies, the sonication can be pulsed or continuous. Pulsed sonication is controlled by multiple parameters, namely, pulse duration, which presents the time between the start and end points of a single sonication, excluding the pause time between consecutive sonication; pulse repetition period (PRP), which represents the time of the pause added to the pulse duration, spatial peak pulse average intensity (ISPPA) representing the averaged intensities of a single Parkinson’s disease, taken over the focal region of the US beam, ISPTA representing the averaged intensities of a PRP, taken over the focal region of the US beam, the duty cycle (DC) which is computed as Parkinson’s disease over PRP, and pulse repetition frequency (PRF), computed from the value of Parkinson’s disease [4,25]. 

Multiple studies have shown US neurostimulation efficacy in AD [26,27]. Zhang et al. reported using ultrasound DBS (UDBS) on APP/PS1 mice associated with an elevation in β-amyloid related to AD, and on aging mice. Telomere shortening was associated with diseases related to aging. So, in the study, Zhang et al. tested the UDBS-treated group (fundamental frequency (ff) = 500 kHz, PRF = 500 Hz, DC = 5%) (*n* = 3) with the sham group (*n* = 3) by analyzing Western blotting of telomerase, which is essential for maintaining telomere length. The author compared UDBS (*n* = 10) versus sham mice groups with respect to Morris water maze (MWM) and fear conditioning tests for both Alzheimer’s and aging mice. Results showed that UDBS could affect the telomer’s length through telomerase activation. Specifically, it was demonstrated that UDBS decelerated the shortening of the telomere in the cortex. Furthermore, mice treated with UDBS showed a significant upregulation of six genes associated with the GABAergic synapse, indicating that UDBS may enhance memory and social behavior through synaptic modulation [26]. Yang et al. used transcranial ultrasound (TUS) with APP/PS1 transgenic mice to investigate its neuromodulator effect on the hippocampus of the deep brain. As in the previous study, spatial memory and cognitive behavior were tested using MWM and fear conditioning tests on the mice. The results indicated an improvement in spatial memory and cognition. Moreover, anxiety levels were also reduced in AD mice who received TUS [27]. 

Low-intensity focused US (LIFUS) enhanced sociability, decreased anxiety levels, and inhibited seizures in epileptic mice [28,29]. A case report of a female subject who suffered from severe treatment-resistant depression and was offered a two-hour LIFUS stimulation session stated that she remained in remission for 44 days as indicated by the fMRI blood-oxygen-level-dependent (BOLD) signal, and Hamilton Depression Rating Score (HDRS-6), which fell from 11 to 0 post-LIFUS treatment [30]. Pulsed US was also tested for its efficacy in treating Parkinson’s disease using MPTP injections in mice. MPTP leads to the accumulation of αSyn protein in the brain, known to halt Parkinson’s disease development if cleared, and inversely, its existence would induce synapse damage and neuronal impairment. In the study, Western blot analysis of a designed wearable US-treated group showed a significant decrease in αSyn, and an immunohistochemistry stain of the neural activity marker ‘c-Fos’ increased with a 30 min US session, indicating ultrasound’s capacity to influence neural signaling as well as its possibility as an effective treatment of Parkinson’s disease [31]. Low-intensity focused US (LIPUS) was shown to facilitate cognition reduction in vascular dementia (VaD)-induced mice. Specifically, LIPUS increased Fndc5, an important cognitive function regulator, and irisin concentrations in aging mice. It was also effective in reducing the number of migraine attacks in mice with nitroglycerin-induced migraine, as reported in [32,33], respectively. A summary of the aforementioned recent studies is shown in Table 1.

### 2.2. Electrical Stimulation

Neuromodulation is a process represented by changes in neural function using one or several stimulation techniques. Electrical stimulation techniques apply electrical currents by selectively stimulating specific areas of the brain region. The non-invasive brain stimulation method known as tES modifies brain function by sending an electrical current through the brain’s cortex. Scientists can monitor changes in behavior by altering the activity of brain areas associated with a particular behavior. Alternating current stimulation (tACS), tDCS, and random noise stimulation (tRNS) are some of the methods used in tES [10,34].

A common, non-invasive technique for modifying brain activity and treating Parkinson’s disease is conventional tES. During electrical stimulation technologies, the central nervous system is affected by controlling neuronal excitability and synaptic transmission. Electrical charges across a neuronal membrane depolarize the membrane and trigger action potential formation as well as the release of neurotransmitters. Neuronal stimulation can enhance or hamper neural activity, depending on the inducing parameters applied throughout the procedure. For example, applying high-magnitude stimulation would lead to the excitation of neural circuits, producing specific responses. In contrast, a low magnitude of stimulation is helpful in inhibiting undesired neural activities. Moreover, long-term electrical stimulation affects the brain’s neural plasticity because it strengthens or weakens the connection through synaptic reorganization [35]. In a study exploring various tES techniques, the finding was that multitarget tDCS targeting both the primary motor cortex and left dorsolateral prefrontal cortex significantly improved the freezing of gait and cognitive performance in Parkinson’s disease patients. The study also noted the potential of tACS and transcranial pulsed current stimulation (tPCS) in modulating brain activity and alleviating symptoms. The parameters for the electric field included 20 min of stimulation per session, with an average electric field strength of 0.25 V/m for tDCS, tACS at varied frequencies such as 77.5 Hz at 15 mA and 20 Hz at 1 mA, and tPCS with pulse durations and inter-pulse intervals of 33.3 µs. These findings highlight the potential of novel tES modalities for improving motor and cognitive functions in PD, though further research is needed to optimize and validate these techniques [35].

Multiple devices that apply an electrical current or stimulation are generally utilized for neuromodulation at clinical and research sites. Fortuitously, like the Omron Electro THERAPY Pain Relief Pro, TENS machines deliver electrical impulses via adhesive electrodes, releasing the pain [36]. SCS systems, such as Abbott’s Proclaim XR, comprise an implanted pulse generator and leads that deliver electrical stimulation to the spinal cord [37]. Similarly, device-based therapies such as the Medtronic Activa PC, which are surgically implanted electrodes and pulse generators, are used to affect deep brain structure movement disorder management [38].

Electrical stimulation techniques offer numerous advantages, including non-invasiveness, reversibility, and the ability to target specific areas of the nervous system. These methods directly interact with neural activity and can be tailored to the patient’s needs. Furthermore, electrical stimulation devices are more affordable and accessible than other neuromodulation approaches. However, there are also some potential drawbacks to consider. Skin irritation, muscle twitching, and pain at the stimulation site may occur as adverse effects. Additionally, responses to electrical stimulation can vary among individuals, and there is limited understanding of the long-term effects on neural tissue [39].

Closed-loop stimulation systems adjust stimulation parameters in real-time based on neural activity feedback, intending to maximize therapeutic output and minimize side effects, adding to patient convenience with wireless, rechargeable devices that require fewer frequent battery replacements. Additionally, the study of non-invasive tES and peripheral nerve stimulation (PNS) methods holds tremendous potential for brain neuromodulation. Research into these breakthroughs continues in efforts to improve further the safety and effectiveness of electrical stimulation used for neuromodulation [40].

### 2.3. Electromagnetic Stimulation

The effectiveness of electromagnetic stimulation in altering brain activity in a non-surgical and selective manner has been widely recognized. This section of the paper delves into the specific technologies utilized in this field, their advantages and disadvantages, device examples, action methodologies, and the latest advancements in this rapidly evolving sector. Magnetic field techniques are utilized by electromagnetic stimulation experts to generate electrical activity in nerve tissue. Transcranial magnetic stimulation is a widely used method that involves the application of brief magnetic pulses to specific regions of the brain [5,41,42].

Scientists are constantly investigating the potential benefits of magnetic fields as a non-invasive method for stimulating the brain [41,42,43,44,45]. Magnetic fields can penetrate the human body with minimal biological impact, making them an intriguing option for delivering DBS without invasive procedures [46]. This approach could avoid any tissue damage caused by implanted devices and offer patients a safer and more effective treatment option [46,47,48]. Although other wireless neuromodulation techniques are being explored [46,49], magnetic fields provide the most precise and extensive access to brain targets, making them an exciting avenue for further research and development in neuroscience [46].

Utilizing magnetic fields is a distinct and robust method for applying biophysical principles. When these fields are directed toward the head, they interact with biological tissues, including the brain, resulting in secondary events [46,47]. These events involve the generation of mild electrical currents and potentially subtle modifications in neurochemical processes. It is significant to recognize that this approach emulates the communication mechanisms already employed by neurons, which depend on chemical and electrical signaling [46]. By enhancing these natural processes, a method such as TMS can regulate neuronal activity outside the brain. This non-invasive method could transform therapeutic interventions for neurological and psychiatric disorders [46,48].

Scientists have researched TMS, a remarkable neurophysiological procedure [41,42,43,44,45] for decades. This NIBS technique has been traditionally employed and has demonstrated significant potential for modulating cortical activity [43,44,50]. In 1985, Barker et al. discovered that a targeted magnetic coil could stimulate the motor cortex and activate specific brain regions to induce contralateral hand movements. This provided critical evidence for the potential of TMS to modulate cortical activity [41,47,50].

Moreover, TMS operates on the principles of electromagnetic induction. By placing a magnetic coil close to the scalp, a rapidly changing magnetic field is generated. This field penetrates the skull and induces mild electric currents in the targeted area of the brain. These currents modulate neural firing patterns, ultimately affecting brain activity. The induced electromagnetic field utilizes the cerebral cortex as a secondary conductor [42,43,46,47].

In the latter part of the 20th century, scientists introduced Paired-pulse TMS, which builds upon TMS. This method (protocol) involves consecutively administering two magnetic pulses through the same coil. The timing between these pulses, the inter-stimulus interval (ISI), is of the utmost importance. Paired pulse TMS can selectively target inhibitory or excitatory connections within the brain by adjusting the ISI to milliseconds or longer. The strength of each pulse and the ISI are essential factors to consider [44].

Furthermore, Paired pulse TMS can investigate the connections between the brain’s two hemispheres (the motor cortex) by delivering pulses to corresponding points. This technique notably effectively identifies inhibitory networks in the brain, which are less understood than the excitatory ones. As such, future research will likely explore these connections further [44,47].

Emerging within the field of NIBS is rTMS. This technique utilizes a coil to generate a pulsed magnetic field penetrating the scalp, akin to the established TMS method. rTMS holds promise as a therapeutic intervention for neurological and psychiatric disorders due to its ability to induce persistent changes in cortical activity beyond the immediate stimulation period. However, recent research suggests the potential for rTMS to induce long-term inhibitory effects along with its excitatory capabilities [44,47].

There are two distinct categories of patterned rTMS: traditional and emerging techniques. Traditional techniques, such as Low-Frequency (LF-rTMS) and High-Frequency (HF-rTMS), are characterized by their stimulation frequency and are believed to either increase or decrease activity in the targeted region of the brain. Meanwhile, emerging techniques, such as Quadri-pulse stimulation (QPS) and Theta-burst stimulation (TBS), are more recent and are currently under investigation. TBS has three key sub-forms: Continuous TBS (cTBS), which has the potential to inhibit; Intermittent TBS (iTBS), which has the potential to excite; and intermediate TBS (imTBS) [44,51].

The technique known as TMS is a precise method for targeting specific brain regions by adjusting the magnetic field. To ensure that the desired muscle area, such as the thumb, responds accurately, the strength of the magnetic pulse is measured, also known as the motor threshold. These pulses can be delivered at specific intervals or in quick succession as a “pulse train,” with the time between each train referred to as the inter-train interval. The rTMS involves repeating these pulse trains, with adjustments made to the speed or frequency of the pulses. Slower frequencies may decrease activity, while faster frequencies may increase it. A form of rTMS called Theta Burst Stimulation (TBS) administers short, high-frequency pulse bursts in a specific pattern, replicating brainwave rhythms. A single TMS session encompasses the entire stimulation period in one day, usually involving multiple sets of pulse trains. Understanding these terms is crucial to comprehending how TMS targets magnetic fields to influence brain activity [47].

It is essential to acknowledge that TMS has become a crucial approach to studying human brain function. Nevertheless, the physiological mechanisms underlying the effects of rTMS and TMS remain incompletely understood. Additionally, the depth of the simulation can range from 2 to 4 cm below the cortical surface, depending on the intensity of the stimulus and the type of coil used. This suggests that only superficial brain structures are being stimulated [44,47]. Ruit et al. have explored the effect of TMS mapping on the interstimulus interval and the number of stimuli needed for reliable motor cortex maps. Using a pseudorandom walk method, the study found that an interstimulus interval of 1.5 s was optimal, balancing participant comfort and data reliability. It recommended a minimum of 80 stimuli to create reproducible maps. A shorter interstimulus interval of 1 s could also be used without compromising map quality if performed by an experienced TMS user. This approach allows TMS maps to be acquired in as little as 2 min, enhancing their feasibility for clinical applications and motor learning assessments. The electromagnetic field parameters included a coil held at a 45-degree angle to the sagittal plane, inducing biphasic currents and stimuli delivered at 120% of the resting motor threshold [52].

While significant research has been conducted on TMS in neuroscience, the technique still faces limitations in precisely targeting specific brain regions and reaching deeper structures [49]. As a result, more robust stimulation of the cortex may be required (which carries some risk) [46], or the development of miniature magnetic coils may be necessary [46,53]. These coils would mitigate safety concerns related to direct electrode contact with neural tissue, which can trigger undesired electrochemical reactions [53].

**Figure 2:**
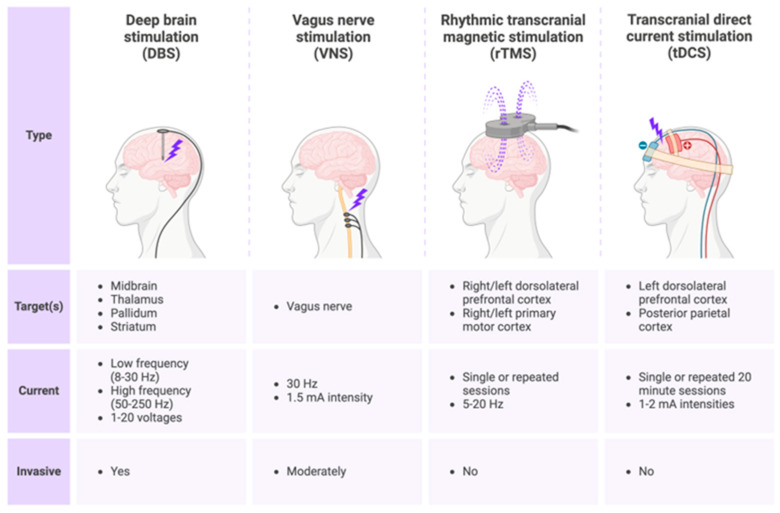
Comparison of brain stimulation techniques for brain disorders, which include DBS, VNS, rTMS, and tDCS. Created with BioRender.com.

The potential of nanomaterial-enabled magnetic neural stimulation has garnered significant attention in both therapeutic and research fields. This innovative approach employs minimally invasive techniques to deliver magnetic nanoparticles to specific brain regions, which can then be activated with magnetic fields to stimulate highly targeted neural activity. This technique promises to treat neurological and psychiatric conditions and revolutionize basic neuroscience research by providing unprecedented precision in studying various brain regions and circuits [49,54,55]. Animal models have already shown promising results, and ongoing research evaluates their safety and benefits for human use [49,54]. The unique advantages of nanomaterials in this technique make them a promising avenue for biomedical applications. Their small size and surface engineering allow targeted delivery to specific cells, enhancing their therapeutic potential. Additionally, their ability to interact with cellular structures with a high degree of precision can open new avenues for treatment and revolutionize the field of biomedical research [49,54,55,56]. Figure 2 illustrates a comparison of brain stimulation techniques for disorders of consciousness.

The underlying principle of these electromagnetic techniques is to induce current within nerve tissue. TMS employs magnetic fields generated and applied to penetrate the skull, stimulating electric currents in the brain’s deep regions beneath the scalp. This depolarizes neurons, leading to controlled action potentials and modulation of the neuronal network. On the other hand, DBS provides natural currents directly to deep brain structures through implanted electrodes, much like a teacher instructing a class. The intensity of these currents may either enhance or inhibit neuronal activity, depending on the stimulation parameters. As a result, targeted brain regions and neural networks can be modulated to regulate activity [5,57]. Mathematical modeling of neural networks can provide significance in understanding complex neural dynamics and have potential applications in the study of dynamic behaviors in neuroscience [58,59].

A wide range of options are available, from tools that generate electromagnetic energy to devices that use electromagnetic fields to treat specific medical conditions. TMS is often conducted in research and clinical settings using devices such as the Magstim Rapid2 or NeuroStar TMS Therapy System. Disposable devices provide precise stimulation parameters for pulse frequency, intensity, and coil orientation, allowing for accurate stimulation of specific brain regions with high reliability [5,57].

Electromagnetic stimulation methods provide some edge over other types of neuromodulation techniques. Besides this, these non-invasive methods significantly reduce the risks and intensity of pain, as they will in invasive methods like surgery. Besides, electromagnetic stimulations offer the exact spatiotemporal control of neural activity needed, along with the localization and manipulation of brain areas and neural circuits. Such a level of precision is quite relevant for research purposes, where researchers can precisely uncover the association between neural activity and behavior by processing biosignals with high accuracy. Besides, these electromagnetic stimulation techniques are readily adjusted to achieve real-time modulation of neural activity and, hence, could be applicable in both experimental and clinical settings [60]. Nevertheless, the electromagnetic stimulation procedures have some hindrances as well. One disadvantage is that the breadth of penetration for magnetic fields in TMS is the limiting factor. Only regions of the superficial brain are stimulated, which does not widen the application of TMS in deep brain structure modulation. Similar to that, DBS involves surgical implantation of electrodes and poses significant risks of infection and tissue damage. Additionally, TMS and DBS are devices that require specialized equipment and experts skilled at utilizing them, preventing these entities from being available in some settings. Further, the long-term consequences of electromagnetic stimulation on neural tissue have yet to be revealed in terms of safety and efficacy. This underlines the requirement for more precise studies to get the answers [60,61].

Recent developments in electromagnetic stimulation technologies have been directed toward enhancing these methods’ safety, effectiveness, and precision. New coil designs allow improved spatial resolution and selective brain region targeting as a result of TMS advancements. Moreover, real-time brain activity mapping and improved stimulation site targeting are made possible by TMS’s integration with neuroimaging techniques (i.e., functional magnetic resonance imaging (fMRI) and electroencephalography (EEG)). Improved positioning of deep brain areas and better treatment outcomes in DBS can be attributed to electrode design and stimulation algorithm advancements. Additionally, ongoing research is being conducted on closed-loop DBS devices, which aim to minimize side effects by dynamically adjusting stimulation parameters in real-time, informed by neural activity. This could improve the effectiveness of treatment.

Electromagnetic stimulation methods present versatile instruments for the selective and time-gated modulation of cerebral activity at a higher level. However, these techniques’ limitations do not diminish the fact that they have shown gradual, if not considerable, progress over the years in the area of research and clinical applications. Continuous breakthroughs in electromagnetic stimulation techniques are increasing their efficacy and safety. Hence, new lines of treatments are unraveled, and knowledge about brain functioning is greatly added [61].

### 2.4. Applications of Neuromodulation Techniques

Neuromodulation techniques have diverse applications in various fields, ranging from managing metabolic disorders and chronic pain at one end to enhancing cognitive function or inducing rapid sleep at the other. The following discusses the multiple uses of non-invasive neuromodulation procedures in managing neuropathic pain, treating movement disorders, treating mental health problems, rehabilitation around neural injuries, and achieving cognitive enhancement through neurofeedback [55].

Neuropathic pain, which is known by the abnormal processing of signals related to pain in the central nervous system and is typically chronic and indiscriminating, is often challenging to handle with conventional treatments since it is not clearly understood. Neuromodulation is an approach to treating neuropathic pain that allows the targeting of dysfunctional nerve tissues responsible for pain processing and, therefore, offers a promising alternative. The application of electrical stimulation methods, such as peripheral nerve stimulation (PNS) and spinal cord stimulation (SCS), has been reported to have a therapeutic effect on neuropathic pain mechanisms involving the re-calibration of pain signals. Additionally, tDCS and TMS significantly lower pain intensity and enhance the quality of life in those with neuropathic pain disorders [55,62].

There are movement disorders that have symptoms like abnormal motor function. These disorders are Parkinson’s disease, essential tremor, and dystonia. They pose a great challenge to conventional medicine, which takes the form of pharmacological treatments. DBS, along with other neuromodulation techniques, has developed as a highly effective therapeutic efficiency that aims to solve movement disorder issues by regulating misleading neural activity in certain brain areas. DBS is the method whereby electrodes are surgically placed into the deep brain structures like the subthalamic nuclei and globus pallidus, respectively, and then powered by an electrical current to stimulate them. The electric current thus activates brain cells, which helps to control the abnormal firing of neurons in movement disorders and hence provides substantial relief in motor symptoms, including tremors, rigidity, and bradykinesia [63].

Non-invasive therapy utilizing neuromodulation techniques has demonstrated efficacy in treating several illnesses, such as depression and substance addiction. For example, DBS and TMS, which have received approval for managing depression, have offered non-invasive and localized approaches to regulate the functioning of affected brain regions associated with these conditions. TMS therapy is often characterized as having the ability to reduce symptoms of depression related to brain conditions that result in either excessive or insufficient communication between nerve cells. Hence, the research findings substantiate the prevailing perspective that transcranial magnetic stimulation can be utilized in conjunction with other therapeutic modalities. Consequently, more symptoms come with mood elevation. Researchers have been concentrating on non-invasive biophysical stimulation techniques, including nerve stimulation (VNS) and tDCS, in the process of successfully treating psychiatric illnesses like anxiety and substance abuse [63].

Transcranial direct current stimulation and functional electrical stimulation (FES), the two electrostimulation techniques, are adopted within rehabilitation programs to stimulate motor recovery, enhance neuroplasticity, and, consequently, have better functional outcomes amongst patients who have suffered a long-term neural injury. The FES implies the conduct of electrical currents to paralyzed or deprived muscles and thus connections with the functioning of electricity in these muscles. Similarly, tDCS operates via stimulation of low-intensity electric currents, focusing on the brain’s cortical excitability and contributing to neuroplasticity. This can help people with neurological injuries improve their motor and cognitive performance. However, innovative techniques, with particular reference to optogenetic stimulation, are on the rise and endeavor to modulate motor management and restoration circuits to improve rehabilitation results [55]. Zhang et al. used a quantum cascade laser to deliver mid-infrared light into mice skulls, showing that mid-infrared modulation (MIM) effectively induces neuronal activation without causing significant tissue heating. Through various experiments, including single-cell electrophysiology and two-photon imaging, they established that MIM activates a substantial number of neurons in targeted cortical regions. Moreover, their results indicate that MIM can significantly boost learning speed by enhancing task-relevant synaptic plasticity, highlighting its potential for applications in cognitive enhancement and brain function modulation. This innovative approach offers a significant advancement over traditional neurostimulation methods, making it a valuable tool for future research and clinical applications [64]. It has been shown that near-infrared (NIR) light, when used with gold nanorods embedded in a gelatin capture structure, induces a photothermal effect that generates localized heat, allowing for the precise and non-destructive release of single cells. This process maintains high cell viability, as demonstrated by cell proliferation assays and live/dead staining. The high temporal and spatial resolution of NIR light enables the selective release of specific cells, making it a powerful tool for isolating individual cells for detailed analysis in biomedical applications [65].

### 2.5. Cognitive Enhancement and Neurofeedback

Neuromodulation treatments offer opportunities in the areas of cognitive enhancement and neurofeedback, which are a category of techniques aiming to improve cognitive functioning and performance or reverse the effects of cognitive deficits. tDCS and TMS are two standard neuromodulation techniques for cognitive enhancement because they use non-invasive and localized stimulation to enhance cortical excitability and improve cognitive functions. These strategies have been demonstrated to enhance multiple brain functions, particularly those related to attention, memory, and executive functions, in healthy people and people with these cognitive impairments. Also, neurofeedback methods involve the real-time monitoring and modulation of brain activity, permitting individualized approaches for improving cognitive function through self-regulation of neural activity patterns. Neurofeedback training programs focused on discrete cognitive abilities, such as attention and memory, have shown effectiveness in increasing cognitive ability and neuroplasticity, reflecting the possible utility of neuromodulation approaches for cognitive enhancement [66].

Neuromodulation methods have a wide range of applications due to diverse domains ranging from managing neurological and psychiatric disorders to accelerating cognitive performance and assisting in rehabilitation after incidents involving neural injuries. These interventions represent adaptive and personalized ways of stimulating the nervous system to promote therapeutic outcomes among patients and improve overall human health in acute and chronic conditions. Continuous research efforts devoted to evolving neuromodulation techniques and explaining underlying mechanisms hold the prospect of broader use of these techniques to treat them clinically and in non-clinical settings at a higher level [66].

### 2.6. Comparison of Ultrasound, Electrical, and Electromagnetic Stimulation Techniques

Neuromodulation can be achieved through various techniques, including ultrasound, electrical, and electromagnetic stimulation. Each method presents its own unique strengths and limitations. Ultrasound stimulation is a promising and innovative approach that utilizes focused sound waves to non-invasively target and modulate specific areas of the brain. It offers exceptional precision and can access deeper brain regions, holding potential therapeutic applications for conditions such as Alzheimer’s, epilepsy, and stroke. Electrical stimulation involves the application of electrical currents to modulate neural activity. This versatile technique is valuable in both research and clinical settings, allowing clinicians to monitor stimulation parameters and address neuronal structures in different neuropsychiatric disorders. Two non-invasive brain stimulation methods using electric currents are tRNS and tACS. These approaches influence behavior and modulate brain activity. tRNS employs random interstimulus amplitudes and intervals, while tACS uses an oscillating electrical current to modify brain activity. In tRNS, pulses are provided over various frequencies and amplitudes dispersed across a specific spectrum range, as opposed to a constant frequency and amplitude in tACS. While electrical stimulation can improve post-injury motion and chronic pain, it has low spatial resolution and faces challenges in selectively targeting individual neuronal assemblies [67].

In contrast, electrostimulation methods, such as DBS and TMS, offer non-invasive and targeted options for neuromodulation. It allows for greater control over brain excitability via magnetic stimulation of specific regions of the brain. This approach has shown potential for both comprehending the functioning of the brain and managing disorders like depression and obsessive-compulsive disorder. Deep brain stimulation involves the placement of electrodes in deep brain structures to regulate neural activity better and improve motor symptoms associated with Parkinson’s disease and essential tremors. Nevertheless, these electromagnetic techniques have limitations regarding resolution and the ability to evaluate brain structures deeply [68]. With this in mind, optogenetic stimulation has revolutionized neuroscience, allowing scientists to explore connections between groups of nerves and their role in shaping behavior. However, optogenetic stimulation requires genetic variation and specialized devices, limiting its use in clinical practice and population-wide experiments [69]. The different brain stimulation techniques are summarized in Table 2.

## 3. The Closed-Loop Neuromodulation System

Neuromodulation, a prevalent technique for investigating the physiology of the nervous system and addressing disorders within it [70], traditionally employs open-loop neurostimulation. However, given the dynamic nature of the nervous system or the targeted organ, the identical stimulus might yield varied outcomes based on the specific physiological state at the time. To accommodate this variability, CLN systems have been developed where stimulation is only delivered in response to specific physiological states or conditions, allowing for real-time adjustments to optimize the therapeutic effects. This approach is particularly beneficial for studying the nervous system, considering the dynamic characteristics of neural activity and how stimuli can elicit various physiological responses depending on the state [70,71]. Recently, neuromodulation has evolved to encompass neural control systems, although traditionally, it refers to the process by which neurons use chemicals to influence the activity of vast neural populations. In clinical settings, closed-loop neuromodulation is a groundbreaking advancement, offering targeted therapeutic intervention for diseases like chronic pain, refractory epilepsy, and Parkinson’s disease, acting as a cerebral pacemaker that detects and counteracts seizure activity, thereby providing intervention only when necessary [71].

### 3.1. The Use of Closed-Loop Neuromodulation (CLN) Systems

Responsive CLN is pivotal in studying neural processes where specific timing between a physiological stimulus and an intervention is crucial. This method allows for two distinct approaches to studying neurostimulation effects: one through an open-loop system, where stimuli are applied across varying states with effects registered separately, and another through a closed-loop system, where stimuli are administered in response to particular states, with effects noted for those states only. Such responsive CLN systems have proven effective in physiological research, particularly in studies of synaptic plasticity [72]. Unlike the open-loop context, where interventions are predetermined and responses measured post-intervention, closed-loop systems define intervention rules and use an automated system to deliver interventions in real-time based on continuous physiological measurements [73,74]. The advantages of on-demand, closed-loop neurostimulation are notable, including a higher likelihood of achieving desired effects and minimizing side effects, along with more efficient stimulus generator operation since stimulation is only administered when necessary. This method has been effectively utilized in several situations, such as using subdural electrodes to stimulate the brain and prevent epileptic seizures [75,76], vagus nerve stimulation (VNS) to control seizure-related heart rate increases [50,77], and DBS to address abnormal brain activity or tremors, demonstrating effectiveness and safety comparable to, or surpassing, open-loop methods while also enhancing the longevity of the implantable generator [77,78].

Furthermore, the need for adaptive neuromodulation is essential when the effects of a neuromodulation intervention are unpredictable, necessitating continuous monitoring to optimize intervention parameters. In physiological research, this is exemplified by the iso-response method, which determines the stimulus-response characteristics of sensory neural circuits. An adaptive CLN system plays a critical role here, recording and quantifying neural activity in real-time to ensure that subsequent stimuli maintain the neural activity within the desired iso-response range. This method streamlines experimental processes and reveals complexities within the stimulus-response function that might otherwise remain hidden [79]. In practice, examples of adaptive CLN systems include VNS for heart rate control. According to Romero-Ugalde et al., the VNS parameters in this system are modified in a manner that aims to minimize the disparity between the recorded physiological variable, precisely heart rate, and a desired goal value of heart rate [80].

Alternatively, closed-loop spinal stimulation attempts to regain movement function in patients with paralysis and alleviate the pain [81,82]. In translational applications, selecting appropriate biomarkers for the adaptation process is pivotal, especially given neuromodulation’s multifaceted and often poorly understood impacts across various organ systems and timescales. Consequently, a meticulous approach to characterizing these effects in animal models and thorough data collection during clinical use are crucial for refining biomarker selection. While clinical applications necessitate a cautious approach to adaptation to mitigate risks, the strategic use of adaptive neuromodulation holds substantial promise for offering tailored, dynamic therapies that respond to the complex interplay of factors influencing treatment outcomes in real-time [83].

### 3.2. Requirements for Closed-Loop Neuromodulation (CLN) Systems

A successful CLN method necessitates the following trio of critical conditions: initially, the target organ’s physiology and the method of the intervention must both have a relatively quick response. The utilization of closed-loop approaches that prioritize prompt action and feedback is unlikely to yield significant benefits for inherently slow processes, such as the utilization of electrical currents for wound healing or bone fracture. Nevertheless, CLN interventions can benefit naturally sluggish processes that depend on rapid physiology, such as rapid synaptic plasticity. These processes encompass those that are regulated by the central or peripheral nervous system, for example, the restoration of motor function in cases of paralysis [82,84,85], the alleviation of chronic pain [81], the suppression of epileptic seizures [86], the enhancement of brain plasticity following neural injury [87], the amelioration of movement impairments in Parkinson’s disease, and even the treatment of psychiatric disorders [86,88].

Second, the feedback signals used in the Clinical Neurophysiology Network system should accurately represent the dynamic state of the target organ. Arm accelerometry is a valuable tool for detecting epileptic seizures, but it may not be as effective for predicting future seizures [75]. Therefore, it is imperative to establish biomarkers that can be accurately measured, monitor the specific physiological process being addressed, and correlate strongly with clinical symptoms and treatment outcomes. This will help in predicting seizures before they become clinically evident [89].

Lastly, the CLN system is recommended over an open-loop system in the pharmacotherapy of human diseases. This is because the latter is nonresponsive and cannot achieve desired effects or cause unwanted effects. Pharmacotherapy can be standardized with a daily dosage regimen that is effective when followed. Nevertheless, interventions are necessary for specific diseases, including diabetes, arrhythmias, hypertension, and asthma, when the circumstances are specific. Closed-loop peripheral neuromodulation is a significant therapeutic method due to the nervous system’s involvement in these diseases. In order to be effective, interventions must be administered under the appropriate conditions, such as airway resistance in asthma, regulation of blood sugar levels in individuals with diabetes, cardiac rhythm management in arrhythmias, and vascular resistance in hypertension [90].

### 3.3. Basic Components of Closed-Loop Neuromodulation (CLN) Systems

A CLN system consists of several components: sensors, a data collection system, a data processing unit, and an output device.

#### 3.3.1. Sensors

Sensors are essential in obtaining physiological data from the nervous system or other body parts because they provide quick reaction times and facilitate repeated testing. The closed-loop system utilizes the physiological data provided by these sensors to deduce the state of the organ or organism. Numerous of these sensors require invasive procedures, necessitating surgical intervention, and are commonly implanted over an extended period of time. The acquisition and the remaining closed-loop systems must be effectively interfaced with each other. The surgical procedures and difficulties linked to persistent, invasive sensors are contingent upon the sensor’s specific type, anatomical placement, and adjacent device. In CLN systems, sensors that detect the electrical impulses of neurons and stimulated cells are considered the most desirable choice since they are cost-effective, widely accessible, and compatible with various amplification and acquisition systems [91].

Closed-loop neuromodulation systems use a range of sensors to monitor neural activity and other physiological signals, including electrical neural activity sensors. Conductive devices, such as microelectrodes, microwires, and pads, are commonly positioned close to the source of activity. According to Lopez-Gordo et al., non-invasive sensors refer to devices used topically on the skin or the surface of the scalp [92]. Nevertheless, most sensors are considered invasive due to their placement in various locations, such as the skin [93], under the skin [94], within the brain [95], deep brain structures [71], the surface of the spine or within the spine [96], or peripheral nerves [97]. The measurement of electrical brain activity offers the primary benefit of achieving a high level of temporal resolution, potentially reaching sub-millisecond scales if necessary. Additionally, spatial resolution can be achieved, however, exclusively with the use of invasive implants with a high number of channels, which consist of microscale sensors. Invasive sensors typically exhibit improved signal-to-noise ratio signals due to their proximity to the electrical signal source, resulting in less ambient noise detection.

Alternatively, sensors may be used to measure the electrical activity of nonneuronal excitable cells, such as electromyography (EMG), electrooculography (EOG), electrocardiography (ECG), and several types of electrodermal activity, comprising the electrostatic skin response [98]. Additionally, invasive sensors like blood flow, temperature, pressure sensors inside arteries or body anatomical cavities, and biochemical sensors for measuring blood pH, CO_2_, and glucose are integral [99]. Beyond these, non-invasive, wearable, and ambient sensors are crucial in CLN systems. The monitoring system tracks several physiological parameters, such as acceleration, respiration, temperature, and oxygen saturation. These are used in ambient sensor systems that utilize light, movement, and video sensing to observe and track a patient’s everyday activities. Using specialized digital acquisition systems and customized CLN system design, these sensors may be connected to different amplification and acquisition systems, expanding CLN systems’ capabilities and effectiveness in the healthcare field [99,100].

#### 3.3.2. Acquisition System

The acquisition system improves and transforms the data from sensors into a digital format, allowing the processing unit to access these digitized signals easily. If the processing unit is situated remotely, the sensors may be integrated or equipped with wireless transmission capabilities [101,102]. Additionally, analog parts, such as amplifiers, can be used to amplify electrical physiological activity and monitor it. Occasionally, analog amplifiers are used to condition signals to fulfill the criteria for digitization [103]. An analog-to-digital converter (ADC) transforms analog signals into digital signals using a suitable sampling rate, precision, and bitrate resolution. Various input signals need differing ADC specifications, with neural spiking activity demanding a more significant sample rate than ECG and BP readings. The acquisition method may also mitigate stimulation artifacts in neural or physiological recordings, which are much larger than physiological signals and result in periods of time without relevant data. Several analog and digital techniques have been created to reduce artifacts. However, this problem has not been wholly overcome [104].

#### 3.3.3. Processing Unit

The processing unit, fundamentally a computer, receives digitized signals as individual samples or packets and conducts real-time operations. Its first function involves executing digital signal processing (DSP) tasks, where digitized signals are further refined to isolate pertinent features. For instance, detecting neuronal spike waveforms from an intracortical microelectrode involves a DSP sequence that identifies spikes based on predefined conditions, such as voltage threshold crossing and comparison with voltage windows. This method similarly applies to ECG and arterial blood pressure signals for extracting specific data points [105]. The second function of the processing unit is integrating various signal features to deduce the system’s physiological state and match it against predetermined conditions to trigger appropriate outputs, termed “intervention rules”. This process is crucial for real-time interactions with the nervous system, particularly in experiments that study the impact of postsynaptic polarization on synaptic plasticity. The third function pertains to the optimization of interventions by analyzing the outcomes of previous ones. The processing unit evaluates the actual response against a desired one, identifying a “response error” and adjusting the intervention parameters to minimize this error in future events, thereby optimizing neurostimulation parameters based on physiological or clinical outcomes [106].

In addition, the processing unit may have local data storage and a wireless module for transmitting data for further analysis. The processing unit’s configuration, which includes both hardware and software, is tailored according to the complicated characteristics of input signals, the intricacies of the digital signal processing (DSP) chain, the flexibility of state estimation and intervention protocols, the frequency of response error calculation, and the intricacy of intervention parameters [107].

#### 3.3.4. Output Device

In the CLN system, the output device executes the intervention. This device is usually a controllable and activating neurostimulator or a system for delivering medication. Neurostimulators are designed to apply targeted energy to neural tissues using various methods, including electrical currents, magnetic fields, ultrasound waves, or light. Such energy can stimulate or inhibit neural activity, thereby inducing physiological effects [108,109]. The primary targets for neurostimulation include peripheral nerves, deep brain structures, the cerebral cortex, and the spinal cord. Energy is transmitted via stimulation probes positioned close to the targeted neural tissues through invasive or non-invasive methods. These probes have similar anatomical and surgical considerations to signal sensors, and many sensors also serve as stimulation probes [110]. The proximity of a probe to neural tissue reduces the energy needed for activation or inhibition, enhancing the specificity and physiological relevance of the modulation. When using invasive neurostimulation sensors, it is essential to carefully analyze the impact of thermal and electrochemical activation to comply with strict safety regulations [111]. Moreover, electromechanical micro-infusion pumps and microfluidic devices serve as delivery methods that can deliver neuroactive chemicals directly to brain tissues via an implanted conduit when activated or configured [110,112].

## 4. Limitations and Future Directions of Non-Invasive Neuromodulation Technologies

In this study, we have discussed various techniques for non-invasive neuromodulation that involve brain stimulation. These techniques include ultrasound, electrical, and electromagnetic fields. These techniques have minimal side effects compared to invasive neuromodulation and typical medication treatments. Moreover, they can be customized for patients who cannot tolerate medication treatment for different reasons. However, non-invasive methods have shown limited effectiveness due to variations in skull thickness, brain patterns, and genetic differences among patients. These factors need to be considered while using these techniques. Additionally, this approach is not suitable for children, pregnant women, and patients with certain medical conditions, which can lead to inconsistent effectiveness across different groups of patients [108].

Ultrasound techniques are a non-invasive neuromodulation approach that offers high spatial precision and is considered safe for deep brain modulation [113,114]. Ultrasound devices are portable and wearable, with multiple arrays of transducers used in research [115]. These devices have undergone continuous development to enhance their suitability for patients in clinical practice. They are compatible with MRI and CT imaging techniques and have been used in clinical trials for animals and humans [116,117,118]. Clinical trials have been conducted using ultrasound techniques to address several brain illnesses, for example, epilepsy, Parkinson’s, and Alzheimer’s diseases [39,118]. Nevertheless, further research is required to assess the enduring effectiveness and safety of the method for patient well-being and other neurological conditions.

Similarly, electrical stimulation techniques offer similar benefits to ultrasound stimulation. These methods are non-invasive, reversible, and can target different brain areas. They interact directly with neural activity and can be customized for each patient. Electrical stimulation devices are also less expensive and easier to obtain than other neuromodulation techniques. However, there are also some drawbacks. Adverse effects such as skin irritation, muscle twitching, and pain at the stimulation site might occur. Furthermore, responses to electrical stimulation can vary between individuals, and there is limited knowledge regarding the long-term consequences for neural tissue [119].

Moreover, electromagnetic stimulation methods provide some edge over other neuromodulation techniques. Besides this, these non-invasive methods significantly reduce the risks and the intensity of pain, as they will in invasive procedures like surgery. Besides, electromagnetic stimulations offer the exact spatiotemporal control of neural activity needed, along with the localization and manipulation of brain areas and neural circuits. Such a technique has a high level of precision on the cell, specifically. It is relevant for research purposes, where researchers can precisely uncover the association between neural activity and behavior by processing highly accurate biosignals [119,120,121,122]. Besides, these electromagnetic stimulation techniques are readily adjusted to achieve real-time modulation of neural activity and, hence, could be applicable in both experimental and clinical settings [60]. Nevertheless, the electromagnetic stimulation procedures have some hindrances as well. One limitation of this technique is that the breadth of penetration for magnetic fields in TMS is the limiting factor. Only regions of the superficial brain are stimulated, which does not widen the application of TMS in deep brain structure modulation. Similar to that, DBS involves the surgical implantation of electrodes and poses significant risks of infection and tissue damage. Additionally, TMS and DBS are devices that require specialized equipment and experts skilled at utilizing them, preventing these entities from being available in some settings. Further, the long-term consequences of electromagnetic stimulation on neural tissue have yet to be revealed in terms of safety and efficacy. This underlines the requirement for more precise studies to get the answers [60,61]. Furthermore, optogenetic stimulation represents a promising approach for more accurate modulation of pain circuits with the characteristics of achieving high spatial and temporal resolution. It also allows one to create personalized pain management strategies like never before [62]. Yu et al. investigate the effects of frequency-specific optogenetic DBS of the subthalamic nucleus (STN) on Parkinsonian motor behaviors in rats. Using an ultrafast opsin called Chronos, the researchers delivered optogenetic stimulation at various frequencies and compared its effects with traditional electrical DBS. They found that high-frequency optogenetic STN DBS (130 pulses per second) effectively reduced pathological circling behavior and improved forelimb stepping, similar to the effects of electrical DBS. The study also highlighted the importance of stimulation rate, as high-rate stimulation produced significant therapeutic effects while low-rate stimulation did not. Additionally, the study demonstrated that optogenetic DBS influenced neural activity by both increasing and decreasing firing rates in the STN, globus pallidus externa, and substantia nigra pars reticulata and suppressed abnormal beta-band oscillatory activity in these regions. These findings suggest that high-rate optogenetic STN DBS can alleviate Parkinsonian symptoms through modulation of neural activity and suppression of pathological oscillations, providing insights into the mechanisms underlying DBS and its potential for treating Parkinson’s disease [123].

## 5. Conclusions

The treatment of brain disorders is experiencing a significant transformation as non-invasive brain sensing and neuromodulation techniques are emerging as powerful alternatives to traditional methods such as invasive surgery and DBS. These techniques, which include ultrasound, electrical stimulation, and electromagnetic stimulation offer, hope for individuals struggling with neurological and mental health conditions. Non-invasive neuromodulation presents promising advantages over invasive brain modulation. The advantage of non-invasive neuromodulation lies in its nature, as it avoids breaking the skin, unlike invasive procedures that carry the risk of infection, bleeding, and long recovery times. This significantly reduces the risk profile and improves patient tolerance. Furthermore, these techniques offer a high degree of customization, as they can target specific brain regions associated with a particular disorder, potentially offering more targeted treatment than medications, which often have widespread effects. However, it is essential to acknowledge the limitations of this evolving field, as skull variations between individuals can affect how these techniques deliver stimulation, potentially impacting their effectiveness. Additionally, inherent differences in brain anatomy and physiology across people can influence how each patient responds to neuromodulation. Furthermore, some techniques struggle to reach deeper brain structures, limiting their application to specific conditions. Further examination is needed to determine the long-term consequences of repetitive neuromodulation on brain tissue since the current research shows promise but does not provide sufficient evidence about the safety of these approaches in the long run. This highlights the importance of continued research and development to refine these techniques and ensure their safe and effective use. Despite these limitations, the potential of non-invasive neuromodulation is undeniable, and with ongoing research and development, we can expect these techniques to become more precise, powerful, and accessible. With this in mind, non-invasive electromagnetic and optogenetic stimulation can offer a promising future for neuromodulation. This paves the way for a future where brain disorders are no longer viewed as untreatable conditions but as challenges that can be addressed through targeted neuromodulation.

## Figures and Tables

**Table 1:** Ultrasound neuromodulation studies in neurodegenerative diseases treatment.

Subject	US Type	US Wave Targeted Brain Region	Targeted Disease	Sonication Parameter	Main Outcomes
Twenty (APP/PS1) male mice Six normal male mice Twenty female aging mice [22]	Focused US	Cortex and Hippocampus	AD	Fundamental frequency = 500 kHz, PRF = 500 Hz, DC = 5%	UDBS could activate telomerase and decelerate telomere shortening, UDBS exerts change on the cortex and hippocampus’s neurons; and it could greatly elevate the cortex’s c-Fos expression. The synapse might be modulated by UDBS and therefore could be related to enhancement of memory and cognition
Twenty-four female (APP/PS1) mice—twelve female control mice—six female APP/PS1 mice for the auditory response experiment [23]	Unfocused US	Hippocampus	AD	Fundamental frequency = 500 kHz, pulse duration = 50 ms, PRF = 1 Hz, DC = 5%, ISPPA = 6 W/cm^2^	TUS enhanced spatial and short-term memory and learning capacity in AD mice and raised AD mice’s epsilon frequency band. Collectively, outcomes indicate that TUS can modulate neural activity and enhance cognitive behavior in AD subjects
Thirty male rats; 14 out of 15 received KA injections and survived, and 15 received non-KA sham injections [24]	Focused low-intensity pulsed US	Striatum ND Hippocampus	Epilepsy	Fundamental frequency = 500 kHz, PRF = 100 Hz, DC = 30%, ISPPA = 1.67 W/cm^2^, ISPTA = 0.5 W/cm^2^	As KA injection leads to hippocampal and striatal volume reduction and higher toxicity, two treatments of focused ultrasound (FUS) partially reversed volume declination. FUS also lowered anxiety levels, and two FUS sessions improved sociability and restored limb balance
23 of 29 male used rats were injected with PTZ for seizure induction. 7 untreated, and 16 treated with LIFUS [25]	Focused low-intensity US	Anteroventral Thalamus and hippocampus	Epilepsy	Fundamental frequency = 1100 kHz, SD = 3 min, PRD = 10 ms, DC = 5%	LIFUS affects GABAergic synapses, therefore leading to the inhibition of seizures in epilepsy subjects
Single female human subject [26]	Focused low-intensity pulsed US	Posterior subcallosal cingulate cortex (SCC), anterior SCC, pregenual cingulate	Depression	Fundamental frequency = 650 kHz, pulse duration = 30 ms, PRD = 4.03 ms, DC = 0.8%	This is a case study of a single human subject with treatment-resistant depression. The modulation of the SCC region assessed using fMRI and the subject’s report of symptom relief for 44 days before relapse suggest ultrasound treatment efficacy in such case
120 male mice; 4 groups: control + sham, MPTP + sham, MPTP + STN + US, MPTP + V1 + US [27]	Pulsed US	Subthalamic nucleus	PD	Fundamental frequency = 3.8 MHz, PRF = 1 kHz, DC = 50%, SD = 1 s, ISPTA = 430 mW/cm^2^	US showed an increase in c-Fos expression in STN and V1 regions, while it is deemed safe as no signs of hemorrhage post-US stimulation were detected. US also greatly improved climbing motor function, reduced proinflammatory cytokines, and suppressed inflammatory signaling in the SN and striatum
Male mice [28]	Focused low-intensity pulsed US	Bilateral hippocampus	Dementia	Fundamental frequency = 1 MHz, pulse duration = 50 ms, PRF = 1 Hz, DC = 5%, ISPTA = 528 mW/cm^2^	In VaD, the hippocampal Fndc5/irisin activity might be corrupted. LIPUS increased Fndc5 expression and irisin concentration in aging mice, and it ameliorated cognitive shortages in VaD-induced mice
56 male rats, eight groups of seven members each [29]	Focused low-intensity pulsed US	No specific brain targets, as stated by the author	Migraine	Fundamental frequency = 500 kHz, SD = 400 ms, PRF = 1 kHz, DC = 50%, ISPPA = 8.3 W/cm^2^	TUS was able to decrease the frequency of migraine attacks but not cerebral blood flow (CBF) velocity

**Table 2:** Overview of the different brain stimulation techniques.

Techniques Type	Ultrasound	Electromagnetic	Electrical
**Spatial resolution**	~1 mm	<1 mm	>1 mm
**Depth of penetration**	10–15 cm or more	Unlimited in theory	5 cm or more
**Testing**	On animals and human	On animals and human	On animals and human
**Cost**	Moderate	High	Low
**Complexity level**	Moderate	Complicated	Moderate
**Reversible**	Yes	Yes	Yes
